# Supplementary material for: Photodynamics of the Molecular Ruby [Cr(ddpd)2]3+
Source: Molecules. 2023 Feb 9;28(4):1668. doi: 10.3390/molecules28041668 (PMC9968007; doi:10.3390/molecules28041668)
Supplement: Supplementary file 1 [file molecules-28-01668-s001.zip › molecules-2199507-supplementary.pdf]

Supporting Information: Photodynamics of the Molecular Ruby $[Cr(ddpd)_2]^{3+}$

J. Patrick Zobel,* Hanna Radatz, and Leticia González*

Institute of Theoretical Chemistry, Faculty of Chemistry, University of Vienna, 1090 Vienna, Währinger Straße 17, Austria.

E-mail: jan.patrick.zobel@univie.ac.at; leticia.gonzalez@univie.ac.at

Contents

S1 Influence IPEA Shift and Active Space of CASSCF/CASPT2 Energies S2

S2 Population of ^{2}E and ^{2}T$_1$ States S5

S3 Fit Modifications of the Excited-State Dynamics Mechanisms S6

S4 Nuclear Dynamics of Additional Internal Coordinates S8

S5 Doublet Minima S10

S6 Additional Supporting Information S11

References S11

S1 Influence IPEA Shift and Active Space of CASSCF/CASPT2 Energies

In order to describe both metal-centered (MC) states and states involving the ligand orbitals, we first constructed an active space comprising the five $3d$ orbitals of the chromium atom as well as two bonding π orbitals and two antibonding π^* orbitals on the ddpd ligands. Using this active space we performed CASSCF and subsequent CASPT2 calculations as described in Section 2.1 in the manuscript. For CASPT2, we tested the influence of the empirical IPEA shift[S1] on the excitation energies of $[Cr(ddpd)_2]^{3+}$. The IPEA shift was introduced to correct for systematic errors found in CASPT2 calculations when describing dissociation processes. The effect of the shift on other properties remains controversial.[S2–S4] Here we show the results of using the IPEA shift with values of $\varepsilon = 0$ and 0.25 a.u. on the excitation energies in Table S1 alongside the CASSCF excitation energies. As can be seen, using the IPEA shift of 0.25 a.u. generally blue shifts the excitation energies by < 0.2 eV compared to using no IPEA shift ($\varepsilon = 0$). Using the IPEA shift of $\varepsilon = 0.25$ a.u. predicts the bright state at 2.71 eV (457 nm) corresponding to the first absorption band centered at 2.85 eV (435 nm), which is in better agreement than using $\varepsilon = 0$, where the bright state is at 2.53 eV (490 nm). The low-lying doublet states are similarly described using both shift values $\varepsilon = 0$ and 0.25 a.u. Thus, due to the overall better agreement of the $\varepsilon = 0.25$ a.u. results with experiment, only these results are discussed in the manuscript.

As our dynamics simulation were planned to include only low-lying metal-centered states, we tested the effect of removing the π and π^* orbitals from the active space on the energies of these states. The results are shown in Table S2. As can be seen, using an IPEA shift of $\varepsilon =$ 0.25 a.u., the differences between the excitation energies using the larger (9,7) active space and the smaller (5,3) active spaces are < 0.1 eV. Thus, it was concluded that this smaller active space could be employed to parametrize the LVC model to simulate the dynamics within the metal-centered states.

Table S1: Excitation energies E in eV and oscillator strengths f from CASSCF (E_{CAS}) and CASPT2 calculations (E_{PT2}) using the (9,7) active space. CASPT2 calculations conducted using IPEA shifts of 0.25 a.u. and 0.00. States are labeled either according to octahedral symmetry, as ligand-centered (LC) states, or as other metal-centered (MC) states. States sorted according to CASSCF energies. Only oscillator strengths larger than 10^{-5} are reported.

State	Configuration	E_{CAS}	$E_{PT2}^{0.25}$	$f_{PT2}^{0.25}$	$E_{PT2}^{0.00}$	$f_{PT2}^{0.00}$
$^4A_2$	$(\pi_1)^2(\pi_2)^2(t_{2g})^3$	0.00	0.00		0.00	
$^4T_2$	$(\pi_1)^2(\pi_2)^2(t_{2g})^2(e_g)^1$	2.38	2.61		2.46	
$^4T_2$	$(\pi_1)^2(\pi_2)^2(t_{2g})^2(e_g)^1$	2.39	2.53		2.35	
$^4T_2$	$(\pi_1)^2(\pi_2)^2(t_{2g})^2(e_g)^1$	2.53	2.71	0.000063	2.53	0.000060
$^4T_1$	$(\pi_1)^2(\pi_2)^2(t_{2g})^2(e_g)^1$	3.55	3.62		3.44	
$^4T_1$	$(\pi_1)^2(\pi_2)^2(t_{2g})^1(e_g)^2$	3.64	3.79		3.65	
$^4T_1$	$(\pi_1)^2(\pi_2)^2(t_{2g})^2(e_g)^1$	3.65	3.78		3.62	
^4LC	$(\pi_1)^1(\pi_2)^2(t_{2g})^3(\pi_2^*)^1$	4.12	4.05	0.000037	3.63	0.000038
^4LC	$(\pi_1)^2(\pi_2)^1(t_{2g})^3(\pi_1^*)^1$	5.22	4.56	0.000010	4.00	
$^4T_1'$	$(\pi_1)^2(\pi_2)^2(t_{2g})^1(e_g)^2$	5.41	5.84		5.75	
$^4T_1'$	$(\pi_1)^2(\pi_2)^2(t_{2g})^1(e_g)^2$	5.62	6.04		5.89	
$^4T_1'$	$(\pi_1)^2(\pi_2)^2(t_{2g})^1(e_g)^2$	5.81	6.31		6.21	
^4LC	$(\pi_1)^1(\pi_2)^2(t_{2g})^3(\pi_2^*)^1$	6.44	6.20	0.000101	5.80	
^4LC	$(\pi_1)^1(\pi_2)^2(t_{2g})^3(\pi_2^*)^1$	6.46	6.25	0.000172	5.65	0.000228
$^4LC/MC$	$(\pi_1)^1(\pi_2)^2(t_{2g})^2(e_g)^1(\pi_2^*)^1$	6.49	6.68	0.000136	6.09	0.000138
$^2T_1$	$(\pi_1)^2(\pi_2)^2(t_{2g})^3$	1.86	1.85		1.84	
^2E	$(\pi_1)^2(\pi_2)^2(t_{2g})^3$	1.92	1.93		1.89	
$^2T_1$	$(\pi_1)^2(\pi_2)^2(t_{2g})^3$	2.01	1.99		1.97	
$^2T_1$	$(\pi_1)^2(\pi_2)^2(t_{2g})^3$	2.03	1.99		1.98	
^2E	$(\pi_1)^2(\pi_2)^2(t_{2g})^3$	2.04	2.01		1.99	
$^2T_2$	$(\pi_1)^2(\pi_2)^2(t_{2g})^3$	3.05	2.91		2.85	
$^2T_2$	$(\pi_1)^2(\pi_2)^2(t_{2g})^3$	3.08	2.92		2.85	
$^2T_2$	$(\pi_1)^2(\pi_2)^2(t_{2g})^3$	3.11	3.02		2.96	
^2MC	$(\pi_1)^2(\pi_2)^2(t_{2g})^2(e_g)^1$	3.96	4.17		3.94	
^2LC	$(\pi_1)^2(\pi_2)^1(t_{2g})^3(\pi_2^*)^1$	4.13	3.66		3.22	
^2MC	$(\pi_1)^2(\pi_2)^2(t_{2g})^2(e_g)^1$	4.23	4.47		4.43	
^2MC	$(\pi_1)^2(\pi_2)^2(t_{2g})^2(e_g)^1$	4.25	4.54		4.44	
^2MC	$(\pi_1)^2(\pi_2)^2(t_{2g})^2(e_g)^1$	4.27	4.59		4.52	
^2MC	$(\pi_1)^2(\pi_2)^2(t_{2g})^2(e_g)^1$	4.28	4.48		4.30	
^2MC	$(\pi_1)^2(\pi_2)^2(t_{2g})^2(e_g)^1$	4.41	4.63		4.31	
^2MC	$(\pi_1)^2(\pi_2)^2(t_{2g})^2(e_g)^1$	4.57	4.92		4.79	
^2MC	$(\pi_1)^2(\pi_2)^2(t_{2g})^2(e_g)^1$	4.72	4.89		4.72	
^2MC	$(\pi_1)^2(\pi_2)^2(t_{2g})^2(e_g)^1$	4.79	5.04		4.91	

Table S2: Comparison of excitation energies E in eV from CASSCF (E_{CAS}) and CASPT2 calculations (E_{PT2}) using the (9,7) active space and (5,3) active space. CASPT2 calculations conducted using IPEA shifts of 0.25 a.u. and 0.00. States are labeled either according to octahedral symmetry.

	Active Space (9,7)			Active Space (5,3)		
State	E_{CAS}	$E_{PT2}^{0.25}$	$E_{PT2}^{0.00}$	E_{CAS}	$E_{PT2}^{0.25}$	$E_{PT2}^{0.00}$
$^4A_2$	0.00	0.00	0.00	0.00	0.00	0.00
$^4T_2$	2.38	2.61	2.46	2.38	2.72	2.67
$^4T_2$	2.39	2.53	2.35	2.39	2.61	2.48
$^4T_2$	2.53	2.71	2.53	2.53	2.70	2.52
$^4T_1$	3.55	3.62	3.44	3.55	3.65	3.47
$^4T_1$	3.64	3.79	3.65	3.64	3.82	3.67
$^4T_1$	3.65	3.78	3.62	3.65	3.81	3.68
^2E	1.86	1.85	1.84	2.25	1.89	1.89
^2E	1.92	1.93	1.89	2.30	1.97	1.94
$^2T_1$	2.01	1.99	1.97	2.39	2.04	2.03
$^2T_1$	2.03	1.99	1.98	2.41	2.04	2.03
$^2T_1$	2.04	2.01	1.99	2.42	2.07	2.05
$^2T_2$	3.05	2.91	2.85	3.44	2.97	2.91
$^2T_2$	3.08	2.92	2.85	3.48	2.98	2.91
$^2T_2$	3.11	3.02	2.96	3.51	3.08	3.03

S2 Population of ^{2}E and $^2T_1$ States

In the manuscript, we have combined the populations of the ^{2}E and $^2T_1$ states into one in order to simplify the analysis. Here in Figure S1 we show the individual populations of both terms separately. As can be seen, both populations follow the same trend throughout all of the simulation time, only differing in their amount that follow the 2:3 ratio of following the number of their components.

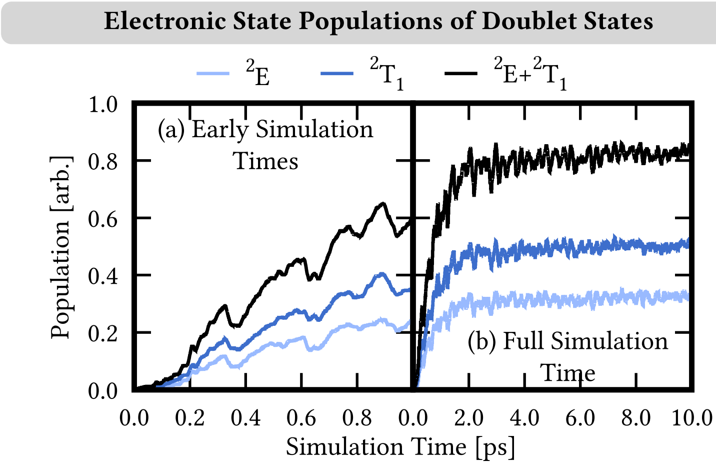

Figure S1: Individual and combined population of the ^{2}E (light blue) and $^2T_1$ (dark blue) electronic states for early simulation times up to 1 ps and the full simulation time of 10 ps (b).

S3 Fit Modifications of the Excited-State Dynamics Mechanisms

As described in the manuscript in Section 3.4, we based the mechanism describing the excited state dynamics of $[Cr(ddpd)_2]^{3+}$ on the analysis of the diabatic character of the trajectories. However, including only the most common reactions observed in the trajectories (Figure 4(c)) did not lead to satisfying fits describing the electronic populations. This is shown in Figure S2(a), where only a minimum number of processes are included in the reaction network. Notable, this leads to a complete quenching of the $^4T_2$ population (orange), a too large increase of the $^2E + {}^2T_1$ population (blue), a too slow decrease of the $^2T_2$ population (green) and a too fast increase of the $^4A_2$ population (violet). Including the ${}^2T_2{}^{cold}$ and ${}^4T_1{}^{cold}$ states (Figure S2(b)) fixes the behavior of the $^2T_2$ and $^2E + {}^2T_1$ populations, whereas the other two problematic behaviors disappear when a second pathway from the $^4T_1$ to the $^2E + {}^2T_1$ states is introduced in Figure S2(c). Introducing instead a $^2E + {}^2T_1$ to $^4T_1$ back-intersystem crossing yields almost the same good description, however with two important differences. First, the fits describing the $^2E + {}^2T_1$ and $^4T_1$ terms become constant after ca. 4 ps in contrast to the simulated time evolution of the electronic state population where population is slowly, but gradually transferred from the $^4T_1$ to the $^2E + {}^2T_1$ states also at later simulation times. Second, the absolute values of time constants of the $^2E + {}^2T_1$ and $^4T_1$ back-and-forth intersystem crossing loose their physical meaning and only the ratio of rate of the processes, amounting only to 1 and 6 fs, respectively. Thus, as the fits in Figure S2(c) reflect best the behavior of the dynamics, only this model is discussed in the manuscript.

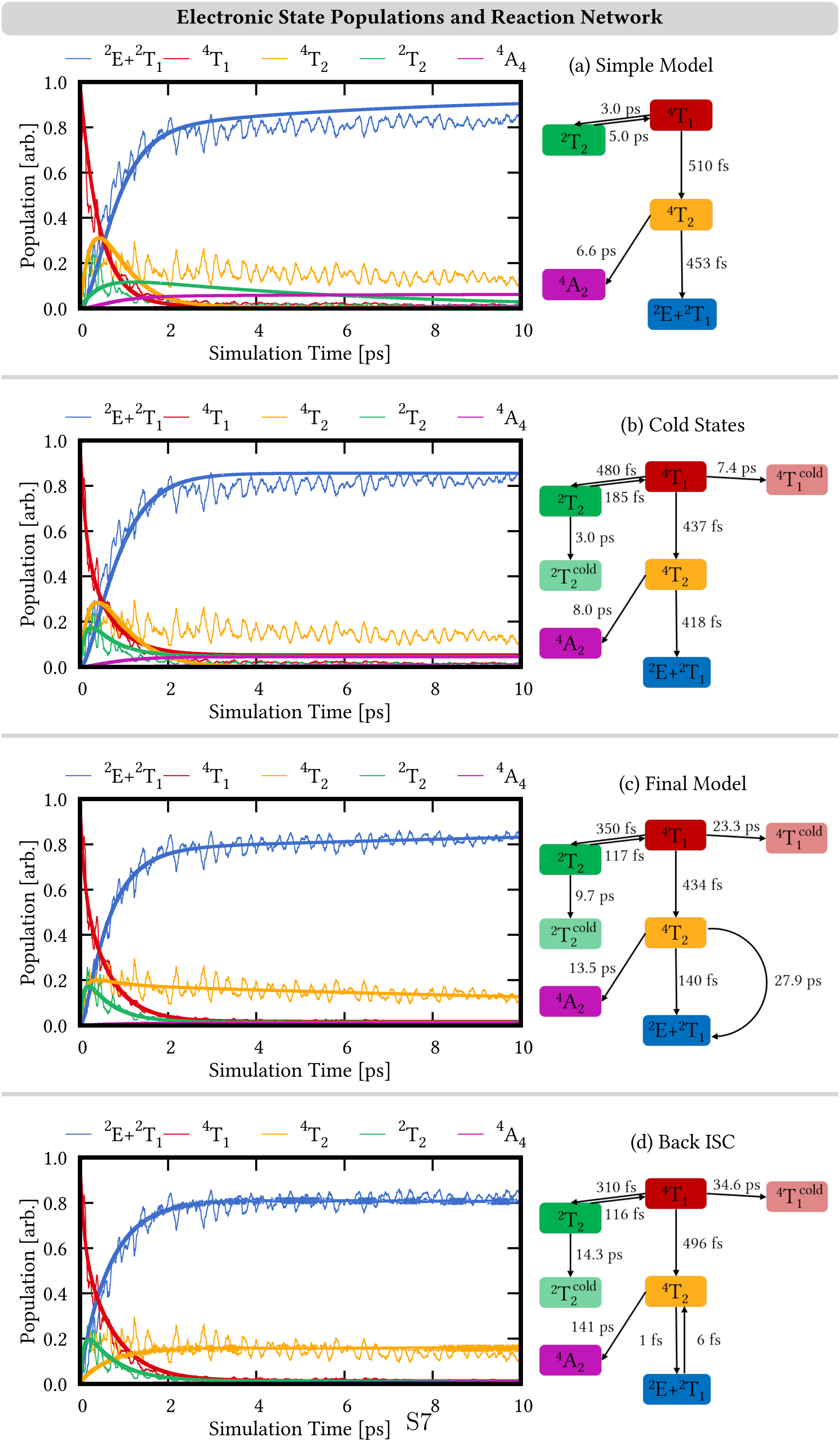

Figure S2: Different reaction networks and fits.

S4 Nuclear Dynamics of Additional Internal Coordinates

In Figure 5 in the manuscript, we show the time evolution of a few internal coordinates that were found to show the largest changes during the dynamics. Here, in Figure S3 we show additional internal coordinates completing the ones shown in the manuscript.

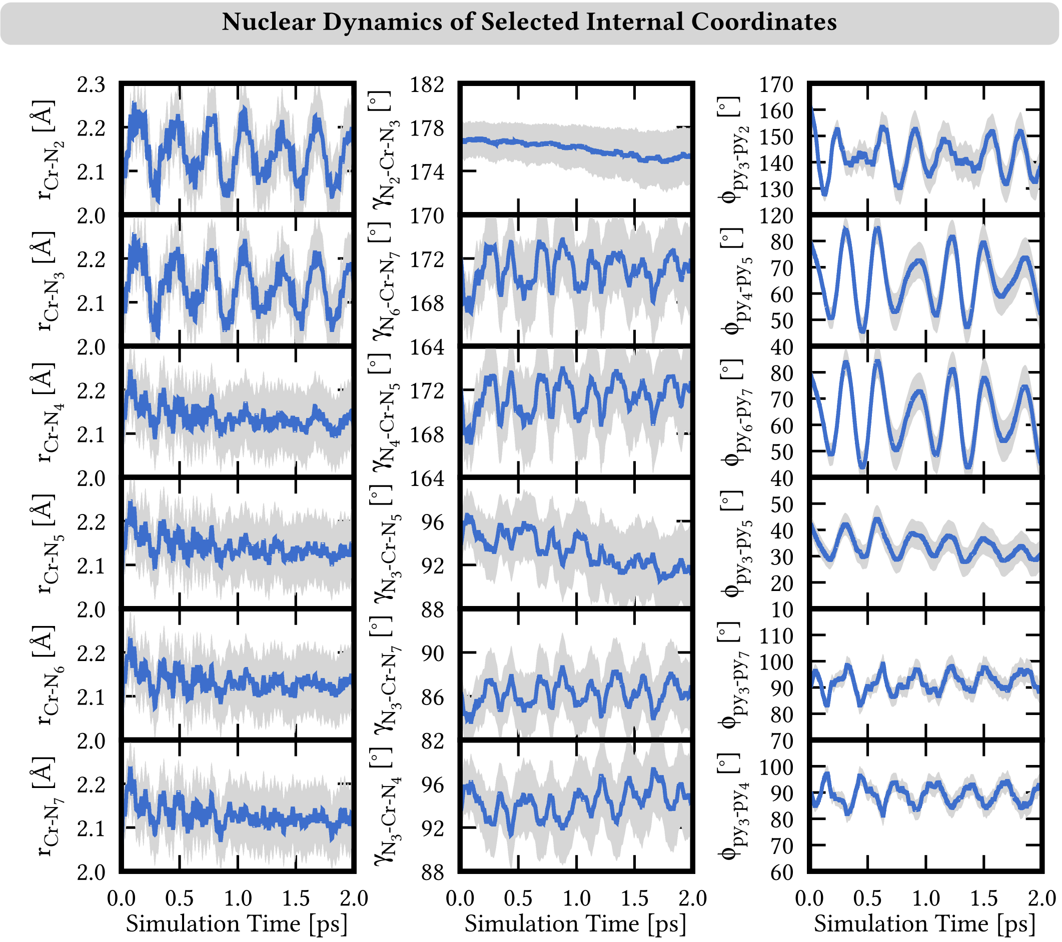

Figure S3: Time evolution of additional internal coordinates. The numbering of the atoms follows Figure 5(a) in the manuscript.

In addition, in Figure S4 we give a pictorial description of the twisting motion of the pyridine rings observed in the dynamics. Figure S4(a) shows the twisting of the central pyridine units py_2 and py_3. These units are rather co-planar at the reference ground-state geometry with an angle of 161°, see structure in Figure S4. During the dynamics, however, the pyridine rings twist against each other towards angles smaller angles following the direc-

tions indicated by the orange arrows. The trans-coordinated peripheral pyridine units, e.g., py_4 and py_5 start at rather perpendicular geometries (81°), see Figure S4(b), while during the dynamics they move towards smaller angles following the motion indicated by the orange arrows.

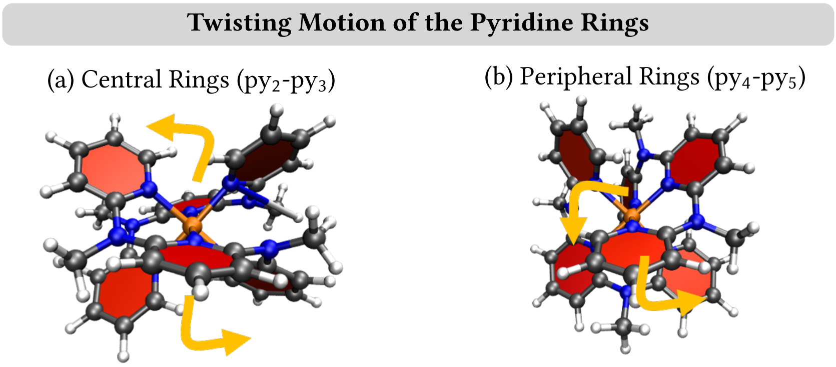

Figure S4: Description of the Twisting Motion of Pyridine Rings

S5 Doublet Minima

As described in Section 3.6 in the manuscript, we have performed geometry optimization for the lowest-excited doublet state on the LVC potential energy surface starting from the ending geometry in the 846 trajectories in a doublet state after 10 ps simulation time. All optimization lead to very similar geometries, and we show a superposition of the optimized geometries together with the averages of a selected internal coordinates in Table S3. Additionally, we present the average excitation energies of the lowest five doublet states at the optimized $D_{1,min}$ geometries in Table S3.

Table S3: Average bond distance r in Å, angles γ and ϕ in degrees, as well excitation energies E in eV from the 846 optimized $D_{1,min}$ geometries. Angles defined as in Section 3.5 of the manuscript. Bottom line: Superposition of ground-state geometry (thick bonds) and 846 optimized $D_{1,min}$ geometries (thin bonds).

$r_{Cr\text{-}N_2}$	$r_{Cr\text{-}N_3}$	$r_{Cr\text{-}N_4}$	$r_{Cr\text{-}N_5}$	$r_{Cr\text{-}N_6}$	$r_{Cr\text{-}N_7}$
2.116 ± 0.002	2.115 ± 0.002	2.113 ± 0.002	2.106 ± 0.003	2.116 ± 0.003	2.093 ± 0.004

$\gamma_{N_2\text{-}Cr\text{-}N_3}$	$\gamma_{N_4\text{-}Cr\text{-}N_5}$	$\gamma_{N_6\text{-}Cr\text{-}N_7}$	$\gamma_{N_3\text{-}Cr\text{-}N_5}$	$\gamma_{N_3\text{-}Cr\text{-}N_7}$	$\gamma_{N_3\text{-}Cr\text{-}N_4}$
174.2 ± 0.4	173.1 ± 0.2	171.1 ± 0.2	89.5 ± 0.4	86.9 ± 0.1	96.2 ± 0.4

$\phi_{py_3\text{-}py_2}$	$\phi_{py_4\text{-}py_5}$	$\phi_{py_6\text{-}py_7}$	$\phi_{py_3\text{-}py_5}$	$\phi_{py_3\text{-}py_7}$	$\phi_{py_3\text{-}py_4}$
142.8 ± 0.6	61.2 ± 0.2	65.6 ± 0.6	126.2 ± 1.9	29.6 ± 0.5	116.9 ± 1.6

$E(D_{1,min})$	$E(D_{2,min})$	$E(D_{3,min})$	$E(D_{4,min})$	$E(D_{5,min})$
1.665 ± 0.001	1.695 ± 0.001	1.741 ± 0.003	1.780 ± 0.001	1.795 ± 0.002

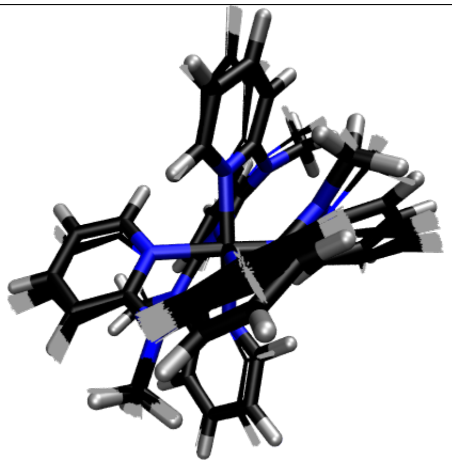

S6 Additional Supporting Information

In addition to this file, the supporting information contains another archive labelled `lvc.zip` including the frequencies and normal modes of the ground-state of $[Cr(ddpd)_2]^{3+}$ (`orca.log.molden`) in Molden format, as well as a file containing the LVC parameters (`LVC.template`) in SHARC format.

References

(S1) Ghigo, G.; Roos, B. O.; Malmqvist, P.-Å. A modified definition of the zeroth-order Hamiltonian in multiconfigurational perturbation theory CASPT2. *Chem. Phys. Lett.* **2004**, *396*, 142–149.

(S2) Zobel, J. P.; Nogueira, J. J.; González, L. The IPEA Dilemma in CASPT2. *Chem. Sci.* **2017**, *8*, 1482–1499.

(S3) Lawson Daku, L. M.; Aquilante, F.; Robinson, T. W.; Hauser, A. Accurate Spin-State Energetics of Transition Metal Complexes. 1. CCSD(T), CASPT2, and DFT Study of $[M(NCH)_6]^{2+}$ (M = Fe, Co). *J. Chem. Theory Comput.* **2012**, *8*, 4216–4231.

(S4) Vela, S.; Fumanal, M.; Ribas-Ariño, J.; Robert, V. On the Zeroth-Order Hamiltonian for CASPT2 Calculations of Spin Crossover Compounds. *J. Comput. Chem.* **2016**, *37*, 947–953.
